# Non-dominant hand use increases completion time on part B of the Trail Making Test but not on part A

**DOI:** 10.3758/s13428-017-0927-1

**Published:** 2017-07-13

**Authors:** Laura Klaming, Björn N. S. Vlaskamp

**Affiliations:** Department of Brain, Behavior and Cognition, Philips Research, High Tech Campus 34, 5656 AE Eindhoven, The Netherlands

**Keywords:** Trail Making Test, B/A ratio, Executive functioning, Non-dominant hand use

## Abstract

The Trail Making Test (TMT) is used in neuropsychological clinical practice to assess aspects of attention and executive function. The test consists of two parts (A and B) and requires drawing a trail between elements. Many patients are assessed with their non-dominant hand because of motor dysfunction that prevents them from using their dominant hand. Since drawing with the non-dominant hand is not an automatic task for many people, we explored the effect of hand use on TMT performance. The TMT was administered digitally in order to analyze new outcome measures in addition to total completion time. In a sample of 82 healthy participants, we found that non-dominant hand use increased completion times on the TMT B but not on the TMT A. The average completion time increased by almost 5 seconds, which may be clinically relevant. A substantial number of participants who performed the TMT with their non-dominant hand had a B/A ratio score of 2.5 or higher. In clinical practice, an abnormally high B/A ratio score may be falsely attributed to cognitive dysfunction. With our digitized pen data, we further explored the causes of the reduced TMT B performance by using new outcome measures, including individual element completion times and interelement variability. These measures indicated selective interference between non-dominant hand use and executive functions. Both non-dominant hand use and performance of the TMT B seem to draw on the same, limited higher-order cognitive resources.

The Trail Making Test (TMT) is a frequently used neuropsychological test to assess aspects of attention and executive functions (Bowie & Harvey, 2006; Lezak, Howieson, & Loring, 2004; Tombaugh, 2004; Wagner, Helmrich, Dahmen, Lieb, & Tadic, 2011). The TMT consists of drawing a trail between elements that are quasi-randomly scattered on paper and has two parts. Part A involves making a trail between numbers in ascending order. Part B consists of 13 numbers and 12 letters, which the patient is instructed to connect in an alternating pattern. The patient is asked to complete the trails as quickly as possible, and completion time is measured as the main outcome (Bowie & Harvey, 2006; Lezak et al., 2004). The clinical interpretation of performance on the TMT is based on part A mainly reflecting visual search and motor speed skills, and part B also requiring higher-order cognitive functions such as cognitive flexibility and task switching (Lezak et al., 2004). The completion time is longer for part B than for part A, and the B–A difference as well as the B/A ratio are considered to reveal deficits in executive function (Arbuthnott & Frank, 2000; Bowie & Harvey, 2006; Corrigan & Hinkeldey, 1987; Gaudino, Geisler, & Squires, 1995; Kortte, Horner, & Windham, 2002; Lezak et al., 2004; Reitan & Wolfson, 1995; Yochim, Baldo, Nelson, & Delis, 2007). Research has consistently shown that performance on the TMT is strongly influenced by age and education, with completion times on both parts of the TMT increasing with growing age and fewer years of education (Amodio et al., 2002; Fromm-Auch & Yeudall, 1983; Robins Wahlin, Bäckman, Wahlin, & Winblad, 1996; Salthouse & Fristoe, 1995; Tombaugh, 2004).

Another factor that may influence TMT performance in a clinical setting is the use of the non-dominant hand. This is an important factor, because patients who suffer from motor dysfunction that prevents them from using their dominant hand may be required to perform the TMT with their non-dominant hand. Currently no norm scores are available for completion times on the TMT with the non-dominant hand, and little is known about how hand use affects TMT performance, which makes it difficult to interpret completion times and derived scores like the B–A difference and the B/A ratio. An increase in completion time or derived score may be falsely attributed to non-dominant hand use, which may result in an underestimation of cognitive problems; alternatively, an increase in completion time or the derived scores may be falsely attributed to a cognitive problem rather than hand use, which would result in an overestimation of cognitive problems.

Hand use may impact TMT performance in several ways. First, research has shown that performance on a motor task is generally slower with the non-dominant hand, which is assumed to be mainly attributable to lower movement accuracy and a greater need for corrective movements (Annett, Annett, Hudson, & Turner, 1979). Research has furthermore shown that healthy individuals are markedly slower when performing a standard neuropsychological test involving fine motor skills, such as copying or cancellation (Cramond, Clark, & Smith, 1989) or name writing (Fromm-Auch & Yeudall, 1983), with the non-dominant hand.

Second, and more problematically, there is evidence for an interference effect between simultaneous performance of motor and cognitive tasks. Studies that have employed a dual-task paradigm have consistently shown that simultaneously performing a motor and a cognitive task increases cognitive load and results in an interference effect, with performance on both tasks deteriorating (Baddeley & Della Salla, 1996; Hausdorff, Yogev, Springer, Simon, & Giladi, 2005; Lindenberger, Marsiske, & Baltes, 2000; Siu, Chou, Mayr, van Donkelaar, & Woollacott, 2008; Theill, Martin, Schumacher, Bridenbaugh, & Kressig, 2012). The interference effect may be stronger when the motor task involves use of the non-dominant hand. For example, healthy individuals were found to perform the recall part of the Rey Osterrieth Complex Figure Test significantly worse when they had used their non-dominant hand as opposed to their dominant hand when copying the figure (Yamashita, 2010). According to the author, this finding is due to drawing the figure with the non-dominant hand leading to fewer cognitive resources being allocated to the performance of the cognitive task—i.e. the copying of the figure. This interpretation is supported by a study that found slowed performance on a cognitive test that requires executive function—i.e. random number generation—when participants simultaneously performed a motor speed task—i.e. the grooved pegboard task—with their non-dominant hand. This dual-task interference effect was not found for the dominant hand (Strenge & Niederberger, 2008).

Imaging research has corroborated the finding that non-dominant hand use interferes with cognitive processing and shown that performing a simple motor task (sequential finger movements) with the non-dominant hand results in greater cortical activity than performing a simple motor task with the dominant hand and in similar cortical activity as performing a complex motor task (random finger movements) with the dominant hand (Mattay et al., 1998). Similarly, another study (Jäncke et al., 1998) has shown that performing a sequential movement with the non-dominant hand (in right-handed subjects) results in greater right hemisphere activation than left hemisphere activation during performance of the same movement with the dominant (right) hand. These findings indicate that motor movements with the non-dominant hand are less familiar and automatic, and therefore consume more cognitive resources.

Taken together, these findings suggest that performing a motor task with the non-dominant hand while simultaneously performing a cognitive task increases cognitive load, which compromises performance on both or one of the two tasks. On the basis of the findings of prior research, we therefore hypothesized that performing the TMT A with the non-dominant hand would not or would only marginally slow down completion times, since there would be little competition for the same cognitive resources because the TMT A mainly reflects visual search and motor speed skills. We furthermore hypothesized that performing the TMT B with the non-dominant hand would increase completion times, since performance of the TMT B requires a substantial contribution of higher-order cognitive functions, and therefore both the TMT B and use of the non-dominant hand would compete for the same limited cognitive resources.

To our knowledge, three studies have explored the effect of dominant versus non-dominant hand use on completion times for the TMT (LoSasso, Rapport, Axelrod, & Reeder, 1998; Toyokura, Ishida, Watanabe, Okada, & Yamazaki, 2003; Toyokura, Sawatari, Nishimura, & Ishida, 2003). LoSasso and colleagues compared completion times on the original TMT and on a parallel version of the TMT for 40 right-handed and 40 left-handed individuals who performed the tests with their both dominant and their non-dominant hand. Completion times were found to be slightly longer for the non-dominant hand for both the original and the parallel TMT B. This intermanual difference was not significant, however, and was considered clinically irrelevant (LoSasso et al., 1998). Toyokura and colleagues (Toyokura, Ishida, et al. 2003; Toyokura, Sawatari, et al., 2003) explored differences in completion times for the Japanese version of the TMT, which consists of numbers (part A) and numbers and Japanese kana letters (part B). In both studies, no intermanual difference in completion times was found (Toyokura, Ishida, et al., 2003; Toyokura, Sawatari, et al., 2003).

Although these three studies have provided interesting insights, a number of important limitations make it difficult to conclude at this time that there is not a clinically relevant difference between performing the TMT with the dominant versus the non-dominant hand. First, completion times were measured manually with a stopwatch in all three studies. Manual measurement of completion times may not always be precise, which may have introduced additional variance unrelated to the participants in these studies. Second, the exact administration procedure of the TMT was not explained in any of these three studies, and it is therefore unclear what the begin and end times of the measurement were and—importantly—to what degree errors have affected completion times. Differences in administration account for the large variability in TMT completion times reported in different studies, which is problematic and is considered to be an important limitation of the TMT (Soukup, Ingram, Grady, & Schiess, 1998; Woods, Wyma, Herron, & Yund, 2015). Treating TMTs with and without errors as synonymous cognitive tests hampers interpretation. Self-correction of an erroneous movement substantially increases the total completion time, which may thus reflect additional motoric and cognitive processes. Errors that are pointed out by the examiner and then corrected by the individual also introduce examiner timing into the completion time. Therefore, trails with errors are different from trails without errors, which makes the inclusion of TMTs with errors in research problematic. It is unclear how many participants made errors on the TMT in the three studies described above and whether these individuals were included in the analyses.

An important limitation of the Toyokura et al. studies (Toyokura, Ishida, et al., 2003; Toyokura, Sawatari, et al., 2003) is that the Japanese version of the TMT was used, which is not directly comparable to the original TMT. The ratio between the TMT A and the TMT B is much higher for the original version of the TMT than for the Japanese version (Toyokura, Sawatari, et al., 2003), indicating that part B is more difficult than part A (Tombaugh, 2004), which makes it impossible to draw any valid conclusions about intermanual differences in completion times for the original TMT based on the two Toyokura et al. studies.

The aim of the present study was to investigate the differences in dominant and non-dominant hand use on the TMT in a sample of healthy individuals. More specifically, we tested the hypothesis that non-dominant hand use would increase completion times on the TMT B but would increase completion times less or not at all on the TMT A. This hypothesis is based on the assumption that non-dominant hand use requires additional cognitive resources, which interferes with performance on the TMT B. In the present experiment, the TMT was administered in the traditional paper–pencil way, but was recorded digitally in an unobtrusive way in order to overcome some of the shortcomings of previous research (LoSasso et al., 1998; Toyokura, Ishida, et al., 2003; Toyokura, Sawatari, et al., 2003) and to be able to explore other measures in addition to total completion time. To our knowledge, this study was the first to look at additional outcome measures, besides total completion time, that could provide valuable information about cognitive functioning and that are not available when administering the TMT without pen digitization. Moreover, this study was the first to look at digitized trails of the traditional paper–pencil TMT.

## Method

### Participants

A total of 117 healthy right-handed individuals participated in the study. Handedness was determined with the Edinburgh Handedness Inventory. One participant was found to have a tendency toward left-handedness (score of –.48) and was therefore excluded from further analyses. The data of 11 participants were not included because of technical issues. Of the remaining 105 participants, 23 (21.9%) made a mistake during either the TMT A or the TMT B or both, and were therefore excluded from further analyses. Participants who made mistakes were excluded in order to obtain a pure measure of total completion time, since as we described above, the correction of errors has a substantial impact on total completion time and changes the test. A mistake was defined as any path that deviated from the correct path—for example, if a participant went from 18 to 20 rather than from 18 to 19. The numbers of participants who made mistakes were similar for both conditions (ten in the dominant hand condition and 13 in the non-dominant hand condition; see below for a description of the conditions). The remaining 82 participants (28 women, 54 men) were all right-handed as measured with the Edinburgh Handedness Inventory (*M* = .80, *SD* = .15). They ranged in age from 20 to 65 years of age (*M* = 36 years, *SD* = 12). Of the participants, 57 (69.5%) had a university degree ranging from a BSc to a PhD, whereas the remaining 25 had a vocational degree as their highest. All participants had normal or corrected-to-normal vision and were employed at Philips Research. Individuals were recruited with flyers and were not compensated for their participation.

### Materials

Participants received the traditional paper–pencil TMT. Underneath the paper TMT, a Wacom Intuos Pro tablet digitized the movements (see also Fig. 8 in the Results). Every new sheet of paper was aligned to markers on the tablet to assure that all TMT’s were performed at the exact same location on the tablet. The position of the paper was fixed by taping it to the tablet. We administered the traditional paper–pencil test to be able to draw conclusions about the TMT as it is currently used in clinical practice. Using a tablet with a screen or a computerized TMT to track movements would not be suitable for this purpose because it would change the physical properties of the test.

The Wacom Intuos Pro tablet sampled the pen position at 133 Hz. Pen positions were recorded with Movalyzer (developed by Neuroscript). Pen pressure was not calibrated and therefore not used in the analyses.

### Procedure

Participants gave written informed consent prior to participation. Participants were randomly assigned to complete the TMT either with their dominant hand (*N* = 41) or with their non-dominant hand (*N* = 41). The TMT was administered in the standard manner, with part A preceding part B. For both parts, the standard practice test with eight items was administered prior to the test.

Participants performed the TMT individually in a room with a research assistant present. The study was conducted by two different research assistants who were trained to use the same instructions for all participants. Administration of the test took approximately 10 minutes.

### Conventional and additional digital parameters for the TMT

Both conventional and additional digital parameters for the TMT were measured and included in the analyses. Conventional parameters include the total completion time, measured both with a stopwatch and digitally as described below. Additional digital parameters for the TMT include a segment-by-segment and element-by-element analysis, interelement variability, and a separation of layout-related processes from executive processes on the TMT B.

#### Completion time

Completion times were determined from the raw pen position data. They were calculated as the difference in time between the moment the pen touched the tablet at the first element and the moment the pen stopped at the last element of the TMT. The pen stop was defined as the velocity of the pen dropping below threshold velocity within 1 cm of the center of the last element. Velocity was calculated as the instantaneous velocity using a second-order polynomial fit to interpolate within a window of five samples centered on the sample of interest. Threshold velocity was adaptively determined through an iterative process for each individual TMT trial to account for noise in the tablet as well as human motor noise. First, a threshold was set arbitrarily. Next, a new threshold was calculated as five times the standard deviation above the mean of the velocity of all samples below the predefined threshold. If the resulting value was lower than the predefined threshold, it was set as the new threshold and a new iteration of the same procedure was executed. This was repeated until the newly calculate threshold was identical to the previous one.

#### Segment-by-segment and element-by-element analysis

Aside from total completion time, we also performed more detailed analyses of the trails. For this purpose, we fully automatically detected the order in which the elements were completed and extracted features per completion. Automatic detection of completion paths was conducted by comparing the spatial pattern within the area closest to an element (or Voronoi cell) with two simple model patterns. One of those model patterns represented completion of the element by connecting the element with the pen entry and exit of the Voronoi cell with two lines (one from the entry into the Voronoi cell to the element and one from the element to the point of exit out of the Voronoi cell); the other model represented no completion with a single line connecting entry and exit of the area. The decision whether an element was completed or not was made on the basis of the similarity of the data to the two models (using a two-dimensional least squares method). If both models made similar predictions (e.g., when an element was completed in a straight path), the decision was made on the basis of whether there had been a local drop in pen velocity close to the element (within a radius of 0.5 cm from the center of the elements). All classifications were then inspected manually; only eight of a total of 4,100 (i.e., 82 times 25 TMT As + 82 times 25 TMT Bs) classifications were incorrect and had to be corrected manually.

An important aspect of TMT performance we were interested in was the time needed to move from one element to the next—i.e. the element completion time. This was defined as the total time required for finding, planning, processing, and executing the movement to the next element. The assumption is that this starts as soon as the preceding element is completed and ends when the target element is completed. We defined the moment of completion as the moment the pen moves with subthreshold velocity as it is approaching the target element. The velocity threshold was calculated in the same way as described above.

With this algorithm for splitting the TMT trail and calculating element completion times, we first explored whether any difference between dominant and non-dominant hand use was related to performance on specific elements of the TMT. We analyzed the data in the same way as Poreh, Miller, Dines, and Levin (2012), who used a computer-assisted version of the TMT. The participants performed the TMT on paper as usual, and with every element completion the experimenter clicked with a mouse on a button that represented the respective element. Poreh et al. divided the TMT A and TMT B into five segments and calculated the time needed to complete each of those segments. They found that the last part of the TMT B is particularly related to executive functioning and hypothesized that this may be related to low search demands, since most elements are cancelled by then. In our study, this translated to the prediction that a difference between the two groups might be particularly evident in the last segment of the TMT B.

We also inspected the trails in further detail on an element-by-element basis to identify any differences between dominant hand and non-dominant hand use. Aside from differences in individual element completion times related to the hypothesis of Poreh et al. (2012), using the left hand might also introduce an effect on individual element completion times because the hand covers different parts of the TMT, which may make it either easier or harder to find certain elements. The element-by-element analysis allowed for an investigation of hand bias of the TMT.

#### Inter-element variability

In the scientific literature and in clinical neuropsychology, there is growing interest in intra-individual variability (IIV; e.g., MacDonald, Li, & Bäckman, 2009; MacDonald, Nyberg, & Bäckman, 2006; Schretlen, Munro, Anthony, & Pearlson, 2003; Strauss, Bielak, Bunce, Hunter, & Hultsch, 2007; Tanner-Eggen, Balzer, Perrig, & Gutbrod, 2015; West, Murphy, Armilio, Craik, & Stuss, 2002). The majority of research on IIV has focused on variability in reaction time tasks, where IIV refers to changes in the reaction time data within an individual on a particular task rather than in the mean reaction time. Reaction time tasks consist of many trials, and because multiple measurements are collected per individual, IIV can be calculated. With the standard paper–pencil TMT there is no way to get insight into the variability in performance within an individual participant, because only the overall completion time is measured. However, with completion times per element derived from the digital pen recordings a measure very similar to the IIV can be calculated, a measure we call the *inter-element variability* (IEV). We derived this measure from the digital pen recordings as follows. First, a distribution was compiled of the completion times per participant for all elements on the TMT except for the first and last elements. Next, the IEV was calculated as the difference between the 10% and 90% cuts through the distribution, as is typically done to calculate IIV. Because the completion times are determined by the participant but also by the element characteristics (e.g., the time needed to find visual information is dependent on its visual eccentricity and on the distance to neighboring information; see, e.g., Vlaskamp, Over, & Hooge, 2005), the element completion times needed to be normalized prior to further processing. Normalization was done by dividing each individual element completion time by the median completion time for each element across participants within a condition. These normalized completion times were then used to calculate the IEV.

Currently in clinical neuropsychology, the mean completion time is a more typical outcome measure. IIV gives an indication about an individual’s consistency across a task or across multiple tasks or sessions, and can therefore provide additional information about the individual’s cognitive functioning. The reason for the growing interest in IIV in the field of neuropsychology is that this measure is considered an informative measure because it is more highly correlated with cognitive dysfunction than is the overall reaction time when patients are engaged in cognitively demanding tasks involving working memory and set switching (MacDonald et al., 2009; MacDonald et al., 2006; Strauss et al., 2007; West et al., 2002). In a clinical context, IIV may provide more insight into the cognitive status of patients and allow for more accurate interpretations of test outcomes (Schretlen et al., 2003; Tanner-Eggen et al., 2015).

#### Separation of layout-related processes from executive processes on TMT B

The TMT B introduces a set-switching task, which causes an increment in completion times relative to the TMT A, which is assumed to be due to increased executive demands. However, performing the TMT also involves other (cognitive) tasks, such as visual search and moving the pen from one element to the next, which are mostly related to the layout of the TMT. Even though the TMT A and B were not designed to differ on these tasks, they do (Gaudino et al., 1995), which leaves open the possibility that an increase in completion time on the TMT B with the non-dominant hand could be due to interference with layout-related processes rather than with executive functions.

Here we sought evidence that non-dominant hand use interferes with executive processes on the TMT B by explicitly separating the contribution of executive processes from the contribution of layout-related processes to the element completion times. For executive tasks, we assumed that the processing time required for the completion of each element of the TMT B was roughly equal (for sake of the argument, we ignore that there might be slight differences between the elements in terms of set-switching; e.g., it may be easier to go from letters to numbers than vice versa, it may be easier to keep the first letters of the alphabet in working memory than later letters, etc.). This means that if non-dominant hand use were to mainly affect executive functions, the same amount of additional processing would be expected for every element completed with the non-dominant hand relative to the processing time for every element completed with the dominant hand. For tasks related to the layout of the TMT (such as visual search and motor tasks), we assumed that a different amount of processing time would be required for each element, depending on its spatial configuration: Some distances between subsequent elements are very long whereas others are short; some elements are in more cluttered areas than others; and sometimes elements are positioned in between subsequent elements. These factors are known to have a large impact on tasks such as visual search (Vlaskamp & Hooge, 2006; Vlaskamp, Over, & Hooge, 2005) and motor processes. On the basis of this assumption, any interference of non-dominant hand use with layout-related processes would be particularly noticeable on elements that already required long processing times with the dominant hand. In short, an across-the-board increment in element completion times in the non-dominant hand condition would be related to executive processes, but systematic increments with longer element completion times in the non-dominant hand condition would be related to layout-related processes.

### Statistical analyses

All statistical analyses were performed using SPSS (version 23). These statistical analyses included independent-samples *t* tests, chi-square tests, and general linear models. Correlation analyses (Pearson and point-biserial) were used to determine the impact of potential covariates.

## Results

We found no differences in age [*t*(80) = –0.365, *p* = .716], handedness [*t*(80) = –0.603, *p* = .548], gender [*χ* ^2^(1, *N* = 82) = 0.868, *p* = .352], and education level [*χ* ^2^(1, *N* = 82) = 1.439, *p* = .230] between the two conditions. The mean completion times for the entire sample for the TMT A were 30.55 s (*SD* = 8.59) measured with a stopwatch and 26.41 s (*SD* = 7.00) measured digitally. This difference was statistically significant [*t*(81) = –10.89, *p* < .0001]. The mean completion times for the entire sample for the TMT B were 52.82 s (*SD* = 13.22) measured with a stopwatch and 48.61 s (*SD* = 12.46) measured digitally, which was also a statistically significant difference [*t*(81) = –8.859, *p* < .0001].

**Fig. 1:**
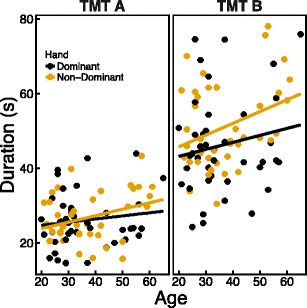
Completion times on the TMT A (left panel) and B (right panel) as a function of age. Each dot represents the completion time of one participant—black dots for participants in the dominant hand condition, and yellow dots for participants in the non-dominant hand condition. The lines are regression lines

In line with previous research (e.g., Tombaugh, 2004), age was found to correlate with both TMT A (*r* = .223, *p* = .044) and TMT B (*r* = .251, *p* = .023) completion times, which increased with increasing age (see Fig. 1). Neither education nor gender correlated with TMT A (education: *r*_pb_ = –.158, *p* = .157; gender: *r*_pb_ = .01, *p* = .926) or TMT B (education: *r*_pb_ = .021, *p* = .853; gender: *r*_pb_ = –.104, *p* = .351) completion times. No differences emerged between men and women in completion times on TMT A [*t*(80) = –0.093, *p* = .926] or TMT B [*t*(80) = 0.938, *p* = .351].

### The effect of hand use on TMT A and TMT B completion times

**Fig. 2:**
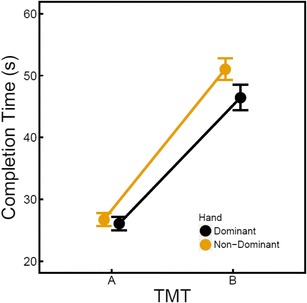
Mean completion times for TMT A and B for the dominant (black dots) and non-dominant (yellow dots) hand conditions. Error bars indicate the standard error of the mean

As can be seen in Fig. 2, the mean completion times for the TMT A were 26.06 s (*SD* = 7.33) for the dominant hand condition and 26.76 s (*SD* = 6.73) for the non-dominant hand condition. The mean completion times for the TMT B were 46.17 s (*SD* = 13.21) for the dominant hand condition and 51.05 s (*SD* = 11.28) for the non-dominant hand condition. The difference between the dominant and non-dominant hand conditions in completion times on the TMT B was on average 4.88 s, which is considerably higher than the difference of 1.9 s found in previous research (LoSasso et al., 1998).

**Fig. 3:**
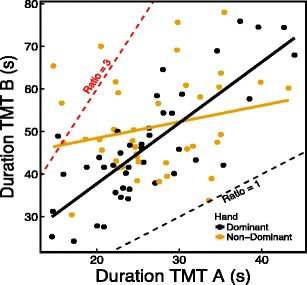
Relation between TMT A and B completion times for the dominant (black) and non-dominant (yellow) hand conditions. The dots indicate individual completion times—black dots for participants in the dominant hand condition, yellow dots for participants in the non-dominant hand condition. The solid lines are regression lines. The red dashed line indicates a B/A ratio of 3, and the black dashed line indicates a B/A ratio of 1

As expected, a general linear model (GLM) with age as a covariate and condition as a fixed factor revealed a main effect of age (*p* = .047) and no difference in completion times between conditions for the TMT A [*F*(1, 79) = 0.141, *p* = .708]. A GLM including TMT B completion time as the dependent variable, age and TMT A as covariates, and condition as a fixed factor revealed an interaction effect of condition and TMT A (*p* = .001). As can be seen in Fig. 3, the TMT A completion time is a good predictor for the TMT B completion time in the dominant hand condition (correlation: *r* = .79, *p* < .0001; slope: *ß* = 1.42), but not in the non-dominant hand condition (correlation: *r* = .229, *p* = .15; slope: *ß* = 0.38). Given the significant interaction effect of condition and TMT A in the model, the TMT A completion time and condition were mean-centered for better interpretability of the model. The GLM revealed a main effect of TMT A completion time (*p* < .0001), a trend for age (*p* = .068), and a trend for condition [*F*(1, 77) = 3.757, *p* = .056]. As can also be seen in Fig. 3, several participants have a B/A ratio score close to or above 3, which is considered a cutoff score for set-switching impairment in clinical practice (Arbuthnott & Frank, 2000). Of the ten participants with the highest B/A ratio scores (all > 2.5), eight were in the non-dominant hand condition and two were in the dominant hand condition.

**Fig. 4:**
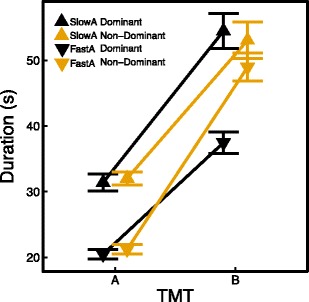
Average completion times for the TMT A and B after applying a median split on the TMT A times (vertex up means slow TMT A, vertex down means fast TMT A). Black lines and symbols represent the dominant hand condition, yellow lines and symbols the non-dominant hand condition. The error bars are standard errors of the mean

We reanalyzed the data after distributing the participants in a slow TMT A group and a fast TMT A group, based on a median split, to better understand the interaction effect of condition and TMT A. The slow TMT A group included 41 participants (dominant hand, *N* = 21; non-dominant hand, *N* = 20), and the fast TMT A group included 41 participants (dominant hand, *N* = 20; non-dominant hand, *N* = 21). As can be seen in Fig. 4, we found a significant difference between the dominant and non-dominant hand conditions in TMT B completion times for participants who had a fast completion time on the TMT A [*t*(39) = –4.125, *p* < .0001], but not for participants who had a slow completion time on the TMT A [*t*(39) = 0.461, *p* = .648]. This difference cannot be explained by demographic factors, since there were no differences in age [*t*(39) = 0.738, *p* = .465], handedness [*t*(39) = 1.226, *p* = .227], and gender [*χ* ^2^(1, *N* = 41) = 0.01, *p* = .92] between the fast and slow TMT A groups in the dominant hand condition, and there were also no differences in age [*t*(39) = –1.396, *p* = .172], handedness [*t*(39) = –0.273, *p* = .786], gender [*χ* ^2^(1, *N* = 41) = 1.336, *p* = .248], and education level [*χ* ^2^(1, *N* = 41) = 0.042, *p* = .837] between the fast and slow TMT A groups in the non-dominant hand condition. In the dominant hand condition, a difference in education level did emerge [*χ* ^2^(1, *N* = 41) = 5.159, *p* = .023] between the fast and slow TMT A groups, with a larger proportion of participants with a higher education in the fast TMT A group, but it seems unlikely that this would explain the difference in completion times on the TMT B. In the fast TMT A group, the difference between the dominant and non-dominant hand conditions in completion times on the TMT B was on average 11.04 s, which is considerably higher than the difference of 1.9 s found in previous research (LoSasso et al., 1998).

### TMT B/A ratio

**Fig. 5:**
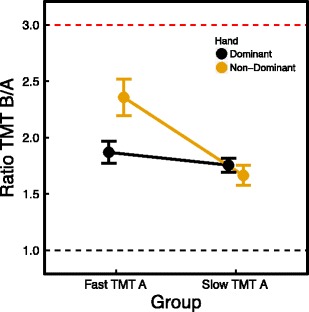
Mean B/A ratios for the fast and slow TMT A groups in the dominant (black dots) and non-dominant (yellow dots) hand conditions. The error bars are standard errors of the mean. The red dashed line indicates a B/A ratio of 3, and the black dashed line indicates a B/A ratio of 1, which means no additional time cost for switching sets on the TMT B relative to the TMT A

TMT B completion times for participants who performed the TMT A fast with their non-dominant hand were markedly different from those for the other groups. Was this also reflected in the B/A ratio? The mean B/A ratio score for the dominant hand condition was 1.8 (*SD* = 0.38), as compared to 2.02 (*SD* = 0.68) in the non-dominant hand condition. A two-tailed independent *t*-test showed that this difference was not statistically significant [*t*(80) = –1.758, *p* = .084]. When dividing the sample into a fast TMT A and a slow TMT A group based on the median split, the difference in B/A ratio scores between the dominant and non-dominant hand conditions was significant in the fast TMT A group [*t*(39) = –2.717, *p* = .01], but not in the slow TMT A group [*t*(39) = 0.635, *p* = .529] (see Fig. 5). In the non-dominant hand fast TMT A group, seven participants (35%) had a B/A ratio score higher than 2.5, as compared to only two participants (9.5%) in the dominant hand fast TMT A group, one participant (4.8%) in the non-dominant hand slow TMT A group, and no participants in the dominant hand slow TMT A group. Put differently, seven (70%) of the participants with a B/A ratio score higher than 2.5 were in the non-dominant hand fast TMT A group.

### Segment-by-segment and element-by-element analysis of TMT completion times

**Fig. 6:**
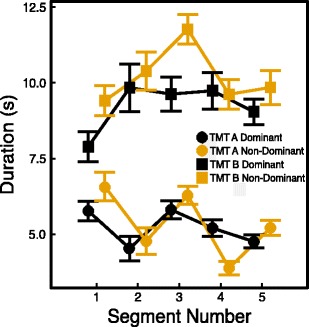
Mean completion times on each of the five segments for the dominant (black symbols) and non-dominant (yellow symbols) hand conditions. Round symbols refer to the TMT A, square symbols to the TMT B. Error bars are standard errors of the mean

To have a more complete understanding of why the differences between the dominant (right) and non-dominant (left) hands occurred on the TMT B, we analyzed completion times over TMT segments as defined by Poreh et al. (2012). Both the TMT A and B were divided into five segments, each consisting of five elements—i.e. Segment 1 consisted of Elements 1 to 5 (A) and Elements 1 to 3 (B); Segment 2 consisted of Elements 6 to 10 (A) and Elements C to E (B); Segment 3 consisted of Elements 11 to 15 (A) and Elements 6 to 8 (B); Segment 4 consisted of Elements 16 to 20 (A) and Elements H to J (B); and Segment 5 consisted of Elements 21 to 25 (A) and Elements 11 to 13 (B). Prior research has shown that on the TMT A, participants are fastest on the first segment and slowest on the third segment, and on the TMT B, participants are fastest on the first segment, slower on Segments 3 and 4, and then faster again on Segment 5 (Poreh et al., 2012). Figure 6 shows the mean completion times per segment for both the TMT A and B for the dominant and non-dominant hand conditions. A mixed analysis of variance (ANOVA) with condition as between-subjects variable and segment as a within-subjects variable showed a significant interaction effect between condition and segment [*F*(2.819, 20.426) = 4.614, *p* = .005] for the TMT A. On the TMT B, a mixed ANOVA showed a significant main effect of segment [*F*(3.534, 54.104) = 5.492, *p* = .001] and no interaction effect between condition and segment [*F*(3.534, 17.802) = 1.807, *p* = .136]. The difference between the two conditions showed a trend [*F*(1, 80) = 3.264, *p* = .075].

These findings suggest that the difference between the dominant and non-dominant hand conditions on the TMT B was due to a general slowing across all segments of the TMT B rather than to a slowing on a specific segment of the test. In the dominant hand condition, participants showed a pattern on the TMT B similar to that found in previous research—i.e. they were fast on the first segment, then slowed down on the second, third, and fourth segments, and accelerated on the last segment (Poreh et al., 2012). In the non-dominant hand condition, participants showed a similar pattern but were particularly slow on the third segment, although the interaction between condition and segment was not significant, as we mentioned above.

**Fig. 7:**
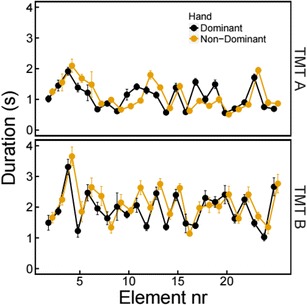
Mean completion times for each element of the TMT A (upper panel) and B (lower panel) for the dominant (black symbols) and non-dominant (yellow symbols) hand conditions. Error bars are standard errors of the mean. Element numbers indicate the order number and do not refer to the content of the elements

To gain an even more detailed understanding, we explored the completion times of the individual elements. In Fig. 7, the mean completion times per element are plotted for the TMT A and B in both conditions. On both parts of the TMT, some elements were completed faster than others, which may indicate that these elements have different physical properties or require different cognitive processes. Moreover, it can be seen that on both parts of the TMT, some elements are completed faster with one hand than with the other.

**Fig. 8:**
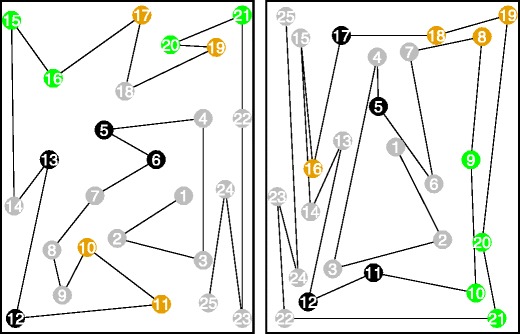
Completion times per element for the TMT A (left) and B (right). Black elements were completed faster with the dominant (right) hand; orange elements were completed faster with the non-dominant (left) hand; green elements had no difference in completion times between the dominant (right) and non- dominant (left) hand

Figure 8 shows the elements with the biggest differences in completion times between the two conditions. The black elements are the ones on which the dominant (right) hand was faster, and the orange elements are the ones on which the non-dominant (left) hand was faster. As can be seen in Fig. 8, for both TMT A and B, all orange elements are on the right of the preceding element, whereas five of the eight black elements are on the left of the preceding element. On the TMT B, the elements that were completed fastest with the non-dominant (left) hand—relative to the dominant (right) hand—were Elements 8 (Segment 1) and 16, 18, and 19 (Segment 4). These are all situated to the right of the preceding element, except for Element 16. Elements 5 (Segment 1), 11 and 12 (Segment 3), and 17 (Segment 4) were completed faster with the dominant (right) hand, and Elements 5, 11, and 12 are clearly to the left of the preceding element.

As was pointed out by LoSasso and colleagues (1998), the hand may block some elements from view and thereby affect TMT performance. This suggestion has not yet been supported by research, but our data seem to confirm that the hand may block some elements from view and thereby affect TMT performance. The locations of the elements with different completion times between the two conditions appear to be systematic to some extent because some elements can be viewed freely with the one hand, whereas they are blocked from view when using the other hand. For some of the elements there is virtually no difference between the two conditions. The differences are smallest for the elements in green in Fig. 8. For the TMT A, these are Elements 15 (Segment 1), 16 (Segment 2), and 20 and 21 (Segment 3), and for the TMT B, these are Elements 9 and 10 (Segment 2) and 20 and 21 (Segment 4).

### Inter-element variability

As we described above, we found that non-dominant hand use affects TMT B but not TMT A completion times. To find further support for the hypothesis that this is due to an interference effect between non-dominant hand use and performance of a task that has high executive demands, we explored performance variability in addition to total completion times by using IEV.

**Fig. 9:**
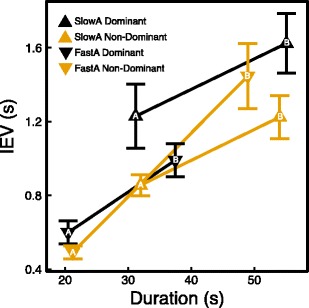
Inter-element variability (IEV) as a function of mean TMT duration. The four lines represent TMT A and B performance for each of the groups. The letters in the symbols indicate the TMT version. The change in IEV from TMT A to TMT B is strikingly different for the non-dominant hand fast TMT A group

In Fig. 9, the mean IEV is shown for the four groups (fast/slow TMT A, dominant/non-dominant hand) on both the TMT A and the TMT B. IEV increases from the TMT A to the TMT B. This was expected, because part B of the TMT requires more executive resources than part A. In addition, it is known from reaction time data that variability increases as reaction time increases (Wagenmakers & Brown, 2007). As can be seen, all slopes are roughly similar, except for the slope of the participants in the non-dominant hand condition who were fast on the TMT A. The slope of the non-dominant hand fast TMT A group is steeper than the slopes of the other three groups [independent samples *t*-test: *t*(78) = –3.217, *p* = .002]. This indicates that for this group, IEV increased more from TMT A to TMT B than in the other three groups, which suggests that the executive load from TMT A to TMT B increased more relative to the other groups. This finding provides additional support for our hypothesis that non-dominant hand use increases the executive demands of the TMT.

### Separation of layout-related processes from executive processes on the TMT B

**Fig. 10:**
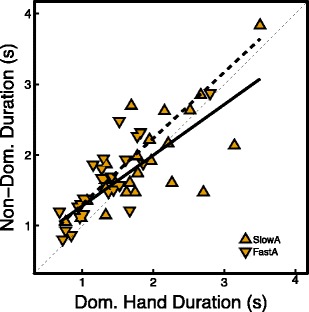
Median completion times for each element of the TMT B with the non-dominant hand, plotted against the median completion time for the same element of the TMT B with the dominant hand. Triangles with the vertex facing down are participants with a fast TMT A, and triangles with the vertex facing up are participants with a slow TMT A. The solid line is a least-squares linear fit to the data of participants with a fast TMT A, and the fat dashed line is a least-squares linear fit to the data of participants with a slow TMT A. The thin dashed line has a slope of 1 and indicates equal performance in the two conditions

Figure 10 shows the median completion times for each element of the TMT B in the non-dominant hand condition, plotted against the median completion times for each element of the TMT B in the dominant hand condition. The plotted times are median times because these are more robust to extreme values than the mean. The two lines show the separate regression lines for participants with a fast TMT A and a slow TMT A. The thin dashed line has a slope of 1 and indicates equal performance in both conditions. By comparing the two regression lines to this line, we can infer whether non-dominant hand use primarily affected layout-related or executive processes. If non-dominant hand use primarily affected layout-related processes, the fitted regression lines would have a slope greater than 1, because completion times in the non-dominant hand condition would go up for elements that required more time to process. This was based on the notion that completion times with the dominant hand would also increase for elements that had higher demands in terms of visual and motor processing. If, on the other hand, non-dominant hand use mainly affected executive functions, the lines would shift upward relative to the dashed line, but the slope would remain 1. This was based on the notion that all elements would have similar completion times in terms of executive processing. As can be seen in Fig. 10, the data are most in line with the latter hypothesis. Both the fast and slow TMT A groups have slopes smaller than 1. In the left part of Fig. 10 the lines are above the dashed line, showing that the non-dominant hand condition was relatively slow on elements with short completion times—i.e. elements that have low visual search and motor demands. In the slow TMT A group, this is averaged out by faster completion of elements with long completion times. In the fast TMT A group, however, the non-dominant hand group is on average slower on elements of the TMT B, independent of their completion times with the dominant hand. The results of this analysis show that the reduced performance with the non-dominant relative to the dominant hand is not due to processes related to the layout of the TMT, but they lend further support to our hypothesis that non-dominant hand use mainly affects executive functions and therefore interferes with TMT B performance.

## Discussion and conclusion

The study has shown that use of the non-dominant hand affects TMT performance. As we hypothesized, hand use was found to increase the completion time on the TMT B but not on the TMT A. This effect was selectively present in a subgroup of participants—i.e. individuals in the non-dominant hand condition who performed the TMT A fast. As a consequence, for this group, non-dominant hand use also affected the B/A ratio; of all participants with a ratio higher than 2.5, 70% were in this group. This finding highlights the importance of a detailed exploration of the data, since participants can show substantial differences in their behavior during a cognitive test like the TMT. On the basis of detailed analyses of the completion times for individual elements of the TMT B—in particular, IEV and an analysis of layout-related processes versus executive processes—we found evidence for our hypothesis that this decrease in performance on the TMT B is related to non-dominant hand use affecting executive functions, thereby interfering with TMT B performance. As we described above, in contrast to the TMT A, which mainly reflects visual search and motor speed skills, completion of the TMT B also requires higher-order cognitive resources. Based on our findings, non-dominant hand use seems to compete for the same limited cognitive resources, which results in a decrease in completion time on the TMT B. We discuss the outcomes and their clinical relevance in more detail below.

### Clinical relevance

It is important to know how non-dominant hand use affects TMT performance, since patients who are unable to use their dominant hand may perform the test with their non-dominant hand. An alternative to administering the written TMT to this clinical group would be to use the oral TMT (Ricker, Axelrod, & Houtler, 1996). It is, however, important to note that the oral TMT has been argued not to be an analogue of the written TMT, but rather a complementary task, because it measures a different underlying cognitive construct (Mrazik, Millis, & Drane, 2010). Moreover, in clinical practice the use of the written TMT with the non-dominant hand seems to be more common than the use of the oral TMT, possibly because psychometric and normative data for the oral TMT are sparse (Mrazik et al., 2010).

Since there is currently limited knowledge about how non-dominant hand use affects performance, the present study provides insights that are highly relevant by clearly showing that TMT completion times and derived scores like the B/A ratio need to be interpreted with caution if a patient uses his non-dominant hand to avoid false attribution of increased completion time and derived scores to cognitive deficits. As the results show, an abnormal test performance may be caused by using the non-dominant hand, which in our study resulted in a mean difference of almost 5 s on the TMT B, which is higher than a difference of 1.9 s found in previous research (LoSasso et al., 1998). A difference of 5 s seems clinically relevant when comparing it to existing norm scores for the TMT (e.g., Tombaugh, 2004). The difference in mean completion time for TMT B between age groups 35 to 44 and 45 to 54 is about 5 s. An increase in 5 s among individuals between 35 and 44 is equal to at least a 10% drop in percentile when scoring in the 30% percentile or better (Tombaugh, 2004). When looking specifically at people who were fast on the TMT A, the effect of using the non-dominant hand becomes even more pronounced. Using the non-dominant hand increased completion time by 11 s in this subgroup. An 11 s increase in completion time on the TMT B is close to the difference in completion time between the age groups 25 to 34 and 45 to 54—i.e. age groups that are 20 years apart. An increase in 11 s among individuals between 35 and 44 is equal to at least a 20% drop in percentile when scoring in the 30% percentile or better (Tombaugh, 2004).

Furthermore, in our sample of healthy individuals, three participants scored on or above the B/A ratio cutoff score of 3 (eight participants had a B/A ratio score higher than 2.5) when they performed the TMT with their non-dominant hand. This was due mostly to a particularly fast completion time on the TMT A and a slow completion time on the TMT B. It seems, therefore, that an abnormal B/A ratio score can be due to hand use and is consequently not a reliable indicator of cognitive deficits if the TMT is performed with the non-dominant hand.

### Digital parameters

In the present study, TMT performance was recorded digitally. The importance of digital measurement of cognitive function has been highlighted by others (Bauer et al., 2012; Poreh et al., 2012; Salthouse & Fristoe, 1995; Schatz & Browndyke, 2002; Woods et al., 2015) because measurements can be done more accurately and in a more standardized way. Moreover, a digital TMT allows for the recording of additional measures that may provide relevant information that is missed in the current paper–pencil version of the test, such as segment-by-segment and element-by-element analysis of the TMT. Research in this area has shown that more detailed analyses of additional parameters can provide valuable information (Poreh et al., 2012; Salthouse & Fristoe, 1995; Woods et al., 2015), which is confirmed by the findings of our study.

Even though our findings show that a slowing in performance on the TMT B with the no-ndominant hand is not due to layout-related processes, the element-by-element analysis revealed that some elements were completed faster than others. It has been hypothesized before that slowing with the left relative to the right hand (and vice versa) on specific elements of the TMT is related to the position of the hand and the fact that the hand obstructs the view of certain elements (e.g., LoSasso et al., 1998). This hypothesis is in line with our data. Generally, elements that are located to the right of the preceding element were completed faster with the left hand and elements that are located left to the preceding element were completed faster with the right hand. It has furthermore been hypothesized that faster completion of the last segment of TMT B is related to a decrease in visual scanning needs and may therefore be a more pure measure of executive functioning (Poreh et al., 2012). Our findings confirm that healthy individuals are faster on the last segment of TMT B. However, since we did not find a difference between the dominant and non-dominant hand condition in completion time on the last segment of TMT B, our findings do not corroborate the hypothesis that faster completion of the last segment is due to a decrease in visual scanning needs and a purer measure of executive functioning.

By looking at the total completion times for TMT A and B, we found support for our hypothesis that non-dominant hand use interferes with performance of the TMT B but not the TMT A because completion of TMT B and non-dominant hand use draw on the same limited cognitive resources. We performed two additional analyses that were possible because we measured TMT B performance digitally. First, we determined the IEV on the basis of individual element completion times. IEV is analogous to IIV in computerized reaction time tasks used in experimental psychology, and it could be an interesting new outcome measure of a computerized TMT. As we described above, there is growing interest in performance variability as an additional outcome measure, since it is more highly correlated with cognitive dysfunction than is the overall reaction time when patients are engaged in cognitively demanding tasks involving working memory and set-switching (MacDonald et al., 2006; Strauss et al., 2007; West et al., 2002). As expected, we found that IEV was higher on the TMT B than the TMT A. Moreover, we found that three of the subgroups showed consistent behavior across the TMT A and B, since their IEV increased equally from the TMT A to the TMT B. The non-dominant hand fast TMT A group, however, showed a larger increase in IEV on the TMT B compared to the other three groups.

We believe the non-dominant hand underperformance on the TMT B is due to motor control and performance of a task that requires executive functions tapping into the same cognitive resources. Non-dominant hand use requires more resources than dominant hand use because the latter is more automatic. Since only limited resources are available, non-dominant hand use can reduce the resources available to perform a task that requires executive functions. As we described above, the TMT B has higher executive demands than the TMT A, and therefore successful completion of the TMT B requires a larger share of the available resources. Participants who were fast on the TMT A used the available resources to enhance their motoric performance. This worked well on the TMT A and made them relatively fast. However, on the TMT B this left too few resources for executive processing, increasing completion time and IEV disproportionally. In contrast, people in the non-dominant group who were relatively slow on the TMT A used the available resources less to enhance their motoric performance, which left room for executive processing when performing the TMT B. This kept the difference between the TMT A and B in completion time and IEV within a normal range.

Besides exploring IEV, we performed a detailed analysis of the completion times for individual elements of the TMT B separating the contribution of executive processes from the contribution of layout-related processes to the element completion times. This analysis clearly showed that the non-dominant hand condition was on average slower on elements of the TMT B independent of the time required for layout-related processes. This finding provides additional support for our hypothesis by showing that non-dominant hand use mainly affects executive functions rather than layout-related processes and therefore interferes with TMT B performance.

As the detailed analyses demonstrate, digital measurement clearly provides the opportunity for exploring the specific underlying processes that contribute to a more complete understanding of how non-dominant hand use affects TMT B completion. In general, we strongly believe that even though at present clinical neuropsychological assessments are conducted in a paper–pencil-based format, it is likely that in the coming years neuropsychological tests will be performed on a digital medium to an increasing extent. It is, however, important to note that although digital neuropsychological assessment offers various benefits, there are a number of important issues to consider, such as the need to establish the psychometric properties of new digital measures (Bauer et al., 2012; Schatz & Browndyke, 2002), the need to understand potential technological complications and limitations (Bauer et al., 2012; Cernich, Brennana, Barker, & Bleiberg, 2007), as well as the need to provide methodological detail regarding computer-based assessment measures to enable replication, which will eventually contribute to confidence in the system and method (Schatz & Browndyke, 2002). We believe that this study contributes to the growing body of research on digital measurement of cognitive function by demonstrating the added value of digital measurement of the TMT.

### Limitations of the study

In line with previous research, we found a correlation between completion time and age (Amodio et al., 2002; Fromm-Auch & Yeudall, 1983; Robins Wahlin et al., 1996; Salthouse & Fristoe, 1995; Tombaugh, 2004). However, in contrast to previous research, we did not find a correlation between completion time and education level. A possible explanation for this finding is that the education level was relatively high in the present study. It is therefore possible that the findings of this study will not generalize to other parts of the population.

Additionally, the sample included only healthy participants. On the basis of the notion that use of the non-dominant hand while performing a cognitively demanding task interferes with its performance because it relies on shared cognitive resources, it seems likely that intermanual differences on the TMT will be even more pronounced in people with cognitive deficits. More research on the underlying mechanisms for individuals whose cognitive functioning is affected as the result of trauma or disease will therefore be necessary.

A third limitation of the study is that the sample consisted only of right-handed individuals. Since our element-by-element analysis suggests that the TMT may be biased for the left or the right hand, it will be important to replicate this study including also left-handed participants.

## Conclusion

The present study has shown that use of the non-dominant hand affects performance on the TMT. Performing part B of the TMT with the non-dominant hand increases completion time, since both using the non-dominant hand and the cognitive task itself draw on the same cognitive resources. Our study hints at important clinical consequences of using the non-dominant hand. A B/A ratio score close to or higher than 3 could be falsely attributed to cognitive dysfunction, whereas at least in some cases a high B/A ratio score may be due to performing the test with the non-dominant hand.

This study demonstrates the importance of a more detailed analysis of TMT performance that is possible when it is measured digitally. A more detailed analysis of the different components of the TMT can be used to better interpret specific outcomes and may eventually be used to improve the reliability of the TMT. The present study therefore adds to the growing body of research on the benefits of digital cognitive testing.
